# How to be a capacitist

**DOI:** 10.1007/s11229-023-04143-0

**Published:** 2023-05-04

**Authors:** Christoph Kelp

**Affiliations:** COGITO Epistemology Research Centre, University of Glasgow, 67-69 Oakfield Ave, Glasgow, G12 8LP UK

**Keywords:** Knowledge, Justification, Perception, Capacity, Knowledge first, Schellenberg

## Abstract

Capacitism is the view that capacities come first in epistemological theorising: they are explanatorily basic and key epistemic phenomena are to be analysed in terms of capacities. This paper develops a problem for capacitism and outlines a motivated way of solving it.

## Introduction

The question as to what comes first in epistemological theorising has enjoyed a considerable degree of popularity in recent literature. One of the most exciting contributions is Susanna Schellenberg’s capacitism (e.g. 2013, 2017, 2018). According to capacitism, capacities come first. They are explanatorily basic in epistemological theorising. Other key epistemic phenomena are to be analysed in terms of capacities.

What makes Schellenberg’s capacitism particularly exciting is that it goes beyond epistemology and uses capacities for theorising across a range of fields, including the philosophy of mind and language. As a result, it is an extremely systematic and rich view, and I could not hope to give it full consideration in this paper. In light of this, I will restrict my focus to the specifically epistemological part of the view. And given the nature of this topical collection, I will do so with a specific eye towards the relation of this view and knowledge first epistemology.

This paper has two central aims: one is critical, the other constructive. The critical aim is to provide reason to think that capacitism and Schellenberg’s specific version of the view remain ultimately unsatisfactory. The constructive aim is to suggest a way forward for the capacitist. In particular, I provide reason to think that a version of capacitism that aligns itself more closely with a knowledge first approach to epistemology than Schellenberg’s carries considerable promise.

Here is the game plan for this paper. Section 2 outlines capacitism as well as Schellenberg’s specific version of capacitism, which she develops for perceptual knowledge. Section 3 raises a general problem for capacitism. In a nutshell, epistemological theorising cannot start with capacities because we need to distinguish between epistemically good and bad capacities and then put epistemically good capacities to use in theorising. I also provide reason to think that epistemically good capacities are capacities that have the function of producing epistemic goods. In Sect. 4, I argue that, as a result, capacitists face a choice concerning the epistemic good that capacities have the function to produce (henceforth ‘the key choice’), with true belief (‘the true belief option’) and knowledge (‘the knowledge option’) as the options. I provide reason to think that both options have drawbacks for capacitists. If they go for the true belief option, they will have to give up their ambitions to analyse justification in terms of knowledge and run into the Gettier problem. On the other hand, if they go for the knowledge option, they won’t be able to hold on to their aim of offering a reductive analysis of knowledge. Section 5 provides reason to think that Schellenberg’s capacitist analysis of perceptual knowledge doesn’t work. While this is a notable critical result in its own right, it also provides some initial reason in favour of going for the knowledge option. Section 6 provides a way forward for capacitists. I outline a distinctive inquiry-centric approach to epistemological theorising and show that this approach provides capacitists with the resources to make their key choice in a motivated way. Finally, in Sect. 7, I use the inquiry-centric approach to provide reason for capacitists to embrace the knowledge option.

## Capacitism and perceptual knowledge

Schellenberg characterises capacitism as the view that capacities are explanatorily basic in epistemological theorising and that other epistemic phenomena such as knowledge, justification and evidence, are to be analysed in terms of capacities. In this way, capacitism is a general epistemological view. Note also that Schellenberg contrasts capacitism with other general epistemological views, including knowledge first epistemology (e.g. Nagel, 2013, Williamson, 2000), evidential internalism (e.g. Huemer, 2007; Pryor, 2000), and reliabilism (e.g. Goldman, 1979; Lyons, 2009), which take other phenomena to be basic in epistemological theorising, to wit, knowledge, conscious mental states, and reliability, respectively (Schellenberg, 2018, pp. 188–189).

While capacitism is a general epistemological view, Schellenberg’s main focus is on epistemological issues of perception. At the very basis of a capacitist approach to the epistemology of perception are perceptual capacities. What are perceptual capacities? Schellenberg’s answer is that they are in essence functional entities. More specifically, her key idea here is that the function of perceptual capacities is to single out and discriminate particulars. Perceptual capacities are individuated by the particulars they have the function to single out. For instance, your perceptual capacity to single out cherries differs from your capacity to single out raspberries in that the former has the function to single out cherries, whilst the latter has the function to single out raspberries (Schellenberg, 2018, p. 38).

In line with capacitism, perceptual capacities are basic and Schellenberg aims to analyse other central phenomena in the epistemology of perception in terms of perceptual capacities. Most importantly for present purposes, perceptual knowledge is analysed in terms of perceptual capacities. Here is Schellenberg’s definition of perceptual knowledge:

- Subject *S* has perceptual knowledge that *p* if and only if *p* is true, *S* employed a capacity to single out what she purports to single out, and *S*’s mental state has the content it has in virtue of *S* having successfully employed her capacity to single out what she purports to single out. (Schellenberg, 2018, p. 206)

To get a better grip on Schellenberg’s definition of perceptual knowledge, let’s look at how it deals with the perhaps two most important kinds of case in the debate on the nature of knowledge, standard cases of knowledge and Gettier cases.

Consider first a case in which I acquire a perceptual belief that there is a dog in front of me. In order for my belief to qualify as knowledge, I must have employed a capacity to single out what I purported to single out. Since in the present case, what I am singling out is a dog, the capacity I employ will have to be a capacity to single out dogs. Had I employed a capacity to single out cats instead, my belief wouldn’t have qualified as knowledge. This is because I would have employed the wrong capacity. What’s more, my belief must have the content it has, i.e. that there is a dog in front of me, in virtue of the fact that I successfully employed a capacity to single out dogs. If it had the content even though the employment of my capacity remains unsuccessful, say because I am merely hallucinating, or if it had the content in virtue of something else, say because I am engaging in wishful thinking, my belief would not qualify as knowledge. Finally, my belief must also be true. If it is not the case that there is a dog before me, then, again, my belief doesn’t qualify as knowledge.

Now consider a standard Gettier case in which I have a justified true belief that falls short of knowledge. To keep things simple, let’s suppose that the thing I am looking at is a robot dog that is so cleverly constructed as to be indistinguishable from a real dog by looking. At the same time, suppose that there is a real dog in front of me, hidden from view by the robot dog I am looking at. In this case, my belief that there is a dog before me falls short of knowledge. According to Schellenberg, the reason for this is that although I employ a capacity to single out dogs, although my belief has its content in virtue of the fact that I employed this capacity, and although my belief is true, I did not *successfully* employ this capacity. After all, the object that I did single out is not a dog and so I didn’t successfully employ a capacity to single out dogs.

With Schellenberg’s account of perceptual knowledge in play, in view of the focus of this topical collection, I’d like to pause at this point to consider the question as to how her capacitism relates to knowledge first epistemology. As a first observation, there are clear differences between capacitism and knowledge first epistemology. Knowledge first approaches start epistemological theorising with knowledge, capacitists starts with capacities. Knowledge firsters typically hold that knowledge does not admit of traditional reductive analysis, capacitism offers precisely such an analysis (at least for perceptual knowledge).

At the same time, there is also an important respect in which capacitism agrees with knowledge first epistemology, to wit, when it comes to the order of explanation between knowledge and justification. This becomes clear in the following passage:

Since perceptual capacities function to single out particulars, their employment yields states that are prone to yield factive evidence and knowledge, even though the environment does not always play along. After all, both the good and the bad case are brought about by employing perceptual capacities. We get at how the world is via perception in a particular way, namely by employing perceptual capacities. And even when we fail to get at how the world is (and so are in the bad case), we are employing perceptual capacities by means of which we aim to get at how the world is. In this way, capacitism provides an explanation of perceptual justification and the way justification is, on the one hand, necessary for knowledge, but why mere justified mental states are nevertheless metaphysically and epistemically dependent on mental states that amount to knowledge. (Schellenberg 2018, pp. 209–10)

According to Schellenberg, perceptual capacities are analysed in terms of a particular type of success, i.e. singling out particulars. In this way, the good case, in which a particular is singled out has explanatory priority. Once we have understood the good case, we can notice that we can employ perceptual capacities not only successfully, but also unsuccessfully. Schellenberg’s view is that perceptual justification corresponds to the employment of perceptual capacities, no matter whether the employment was successful or unsuccessful. By way of illustration, let’s return to our Gettier case once more. Here my belief that there is a dog before me is not knowledge. In particular, I do not single out a dog, which means that I do not successfully employ my perceptual capacity to single out dogs and so do not know that there is a dog before me. Even so, the thought is that I employ my perceptual capacity to single out dogs. That, according to Schellenberg, is why my belief that there is a dog before me is nonetheless justified.

The key point for present purposes is that, just like knowledge firsters, Schellenberg take perceptual knowledge to have explanatory priority over perceptual justification. This is an important point of agreement.

## A problem for Capacitism

With these points about capacitism in play, in what follows, I will develop a problem for the view. I’d first like to take a closer look at capacitism as a general epistemological view and, in particular, at the difference between capacitism and knowledge first epistemology. Recall that capacitism is a general epistemological tenet according to which capacities are explanatorily basic in epistemological theorising. In contrast, according to knowledge firsters, it is knowledge that is thus basic.

To begin with, note that even if the view can be made to work for the epistemology of perception, there is reason to doubt whether it works equally well as a general epistemological view. In short, this is because not all capacities are capacities to produce epistemic goods such as knowledge and true belief. The capacity to engage in wishful thinking is one example, the capacity believe in the face of the evidence is another.^1^ In this way, there is reason to think that capacities cannot be what’s explanatorily basic in epistemological theorising. What is needed in addition is a way to distinguish epistemically good capacities (such as perceptual and inferential capacities) from epistemically bad capacities (such as the capacity to engage in wishful thinking). If this isn’t immediately obvious, consider what happens if we continue in the spirit of capacitism and use capacities to analyse key epistemic phenomena. To take just one example, consider the following generalisation of Schellenberg’s analysis of perceptual knowledge: S knows that p if and only if S employed a capacity and S’s belief has the content it has in virtue of having successfully employed her capacity. If the capacity is the capacity to engage in wishful thinking, we get the result that beliefs acquired via wishful thinking can qualify as knowledge. Since this result is clearly unacceptable, we’ll do well to restrict the capacities in terms of which we analyse key epistemic phenomena such as knowledge to epistemically good capacities.

What are epistemically good capacities? Recall that capacities are functional entities, i.e. they do things. Accordingly, one dimension along which we can assess capacities concerns how well they do what they have the function of doing. Consider knives, which have the function of cutting. Some knives are better than others because they cut better. Likewise, your capacity to hit targets in archery may be better than mine because it is more reliable. However, this dimension of assessment doesn’t help with the question we are interested in, which is whether a certain capacity is epistemically good. More generally, it doesn’t help with the question of whether a certain capacity is good relative to some kind of good, such as epistemic, moral, practical, etc. good. For instance, the fact that your capacity to hit the target in archery is more reliable than mine leaves open the question of whether capacities to hit the target in archery are morally, practically, epistemically etc. good capacities. What matters here is whether what the capacity has the function of doing is to produce some good of the kind in question. For instance, your capacity to make everyone you interact with happy is a morally good capacity. And the reason for this is what the capacity has the function of doing, i.e. making people happy, is a moral good. In contrast, your capacity to hit targets in archery is not a morally good capacity. While you may use your capacity for moral good, you may also use it for moral bad. As a result, your archery capacity is neither morally good nor morally bad. But, of course, if what matters to whether a capacity is good relative to some kind of good is that what it has the function of doing is to produce the kind of good in question, then what it takes for a capacity to be epistemically good is for it to have the function of producing some epistemic good. Note also that this allows us to explain why the capacity to recognise dogs is an epistemically good capacity and the capacity to engage in wishful thinking isn’t. The former is a capacity to produce an important epistemic good, such as perceptual knowledge or perceptual true belief about dogs. In contrast, the latter isn’t.

Crucially, since epistemically good capacities are capacities that have the function of producing some epistemic good, capacities cannot be explanatorily basic in epistemological theorising. This is the first critical result of this paper. Rather, what must at the very least be more basic are the epistemic goods that serve to distinguish good epistemic capacities from bad ones. The direction of explanation will go from these epistemic goods to epistemic capacities. This is the paper’s first constructive point.

## The key choice

What are the epistemic goods in terms of which good epistemic capacities are distinguished for bad ones? Here capacitists faces an important choice point. They can either embrace the knowledge option and side with knowledge firsters in holding that the epistemic good in question is knowledge. Alternatively, they can go for the true belief option and hold that it is true belief.^2^ This is what I earlier called the key choice for capacitists.

There is evidence that Schellenberg takes the epistemic good in question to be knowledge. This is because she associates function fulfilment for capacities with knowledge:

Perceptual knowledge differs from mere justified true mental states in that the capacities employed in knowledge in fact succeed in serving their natural function. In mere justified true mental states, the capacities are employed while failing to single out what the subject purports to single out. (Schellenberg 2018, p. 208)

But, of course, if capacities are functional entities and function fulfilment is associated with knowledge in this way, the epistemic good that makes capacities epistemically good is knowledge. In this way, there is reason to think that Schellenberg sides with knowledge firsters on this count.

Unfortunately, things are more complicated. To see why, note that if the epistemic good we are after is indeed knowledge, then the good epistemic capacities in question are capacities to know. But if that is so, then the project of offering a reductive analysis of knowledge in terms of capacities is bound to fail.

Now, Schellenberg considers this issue and makes it clear that she does not take the capacities at issue in her analysis of perceptual knowledge to be capacities to know. Here goes:

Now, one might argue that insofar as employing perceptual capacities yields knowledge, these capacities should simply be analyzed as capacities to know. But such a view would put the cart before the horse. It is unclear what the explanatory gain would be of analyzing knowledge in terms of capacities to know. Arguably, any such account would be circular. (Schellenberg 2018, p. 209)

It is worth noting that this does not mean that Schellenberg accepts an analysis of perceptual capacities in terms of true belief. Rather, she offers an alternative answer in the following passage:

According to capacitism, the perceptual capacities in play are not analyzed as capacities to know: one neither employs the capacity to know when one is in the bad case, nor when one is in the good case. Perceptual capacities are analyzed rather in terms of their natural function, namely their function to discriminate and single out particulars in the environment. (Schellenberg 2018, p. 209)

This passage suggests that Schellenberg takes the epistemic good that perceptual capacities are explained in terms of to be neither knowledge nor true belief, but rather discriminating and singling out particulars in the environment.

Now, I do not mean to contest this claim about perceptual capacities. What I’d like to ask about instead is how this view generalises from perceptual capacities to epistemically good capacities in general. To see why this is a fair question to ask, recall that capacitism is a general epistemological view.^3^ This means that any claim advanced as part of capacitism in the epistemology of perception in particular must generalise beyond the epistemology of perception to general epistemology. As a result, Schellenberg’s claim that perceptual capacities are explained in terms of their function to discriminate and single out particulars in the environment must generalise to general epistemology. The question is how it might do so.

As a first observation, note that it doesn’t generalise in the most straightforward way, i.e. that capacities (or at the very least epistemically good capacities) in general have the function of discrimination and singling out particulars in the environment. To see this, note that other epistemically good capacities cannot plausibly be thought to have this function. Testimonial and inferential capacities are perhaps the clearest examples here.

This leads us back to the question as to how else Schellenberg’s claim about perceptual capacities might generalise. Here is the best answer I can think of. If the function of perceptual capacities qua epistemically good capacities is to single out and discriminate particulars in the environment, this must be because singling out and discriminating particulars in the environment is an instance of a more general epistemic good that is also realised in other epistemically good capacities such as testimonial and inferential ones. But now the question that arises is what the more general epistemic good might be. And here we are clearly back to our original contenders, i.e. knowledge and true belief.

One might wonder whether this is of any concern for Schellenberg. The reason why the answer is yes is that it is this more general level of abstraction that is key in general epistemology. It is the one at which the general analysis of knowledge, of which Schellenberg’s analysis of perceptual knowledge is an instance, will be found. But if it turns out that, at this general level, the epistemic goods in terms of which epistemically good capacities are explained is knowledge, then epistemically good capacities at this general level will be capacities to know. In that case, any reductive analysis of knowledge in terms of epistemically good capacities is going to be circular. The problem for a reductive analysis of knowledge cannot be circumvented in the way envisaged by Schellenberg after all.^4^

So, perhaps then a better option for capacitists is to go with true belief as the epistemic good in terms of which epistemically good capacities are explained rather than knowledge. This will bring the prospects of a reductive analysis of knowledge back into view. After all, the direction of analysis can now proceed in the familiar, traditionalist way, from true belief, to justification, to knowledge.

At the same time, there are also drawbacks here. First, if capacitists go down this route, the envisaged account of the explanatory relation between knowledge and justification will no longer work. Capacitism will no longer deliver an account on which justification is explained in terms of knowledge. After all, the central capacities are explained in terms of their function of producing true beliefs. And if justification is explained in the way envisaged in terms of the employment of capacities, justification isn’t explained in terms of knowledge.

One might wonder whether this really is a concern. Unfortunately, there is reason to think that it is. To see why, note first that it puts the capacitist account of Gettier cases in jeopardy, which very clearly relies on the idea that knowledge enjoys explanatory priority over belief. Schellenberg takes it that cases of perceptual knowledge are cases of function fulfilment and Gettier cases, i.e. cases of justified true belief that fall short of knowledge, aren’t (Schellenberg, 2018, p. 208). It is easy enough to see that this explanation will no longer work on the present proposal. After all, if the epistemic good in question is true belief, then perceptual capacities will fulfil their function when they produce a true belief. As a result, Gettier cases will be cases of function fulfilment and the envisaged explanation will no longer work.

- Relatedly, the Gettier problem (e.g. Gettier 1963, Lycan 2006) will start looming for Schellenberg. To see this, consider the following Gettier case. You are looking at a lump of gold that, unbeknownst to you, has a coating of fake gold that is indiscriminable from real gold by perception alone. You form a true belief that the lump is gold. On the present proposal, your belief does meet the capacitist conditions for perceptual knowledge. After all, you did employ a capacity to single out what you purported to single out, i.e. gold, and your mental state has the content it has, i.e. that the lump is gold, in virtue of you having successfully employed your capacity to single out what you purported to single out. The key reason why you satisfy this condition is, of course, that, on the present proposal, what it takes to successfully employ your perceptual capacity to single out gold is unpacked in terms of true belief (rather than knowledge). Since the belief you form is true, you did indeed successfully employ your perceptual capacity. And of course, your belief has the content it has, i.e. that the lump is gold, in virtue of you successfully employing your perceptual capacity. As a result, the capacitist account of perceptual knowledge predicts that your perceptual belief qualifies as knowledge. At the same time, since the case is a standard Gettier case, the account runs into the Gettier problem.^5^

Recall that Sect. 3 argued that capacities cannot be explanatorily basic because we need a way to distinguish epistemically good capacities from epistemically bad capacities. Instead, if we want to put capacities to use in epistemological theorising, we will do well to start with some epistemic good in terms of which these capacities are analysed. This section has focused on the question as to what this good is. The two options are that it is knowledge and that it is true belief, and, in this way, the key choice comes into clear view. The second critical result of this paper is that both options have serious drawbacks. If capacitists go for the knowledge option, the prospects of a reductive analysis of knowledge start looking dim. If they go for the true belief option, the envisaged analysis of justification in terms of knowledge runs into trouble, and the Gettier problem starts looming.

## A problem for Schellenberg’s analysis of perceptual knowledge

In this section, I want to argue that there is independent reason to think that Schellenberg’s analysis even of perceptual knowledge remains ultimately unsatisfactory. Now, this is an interesting critical result in its own right. That said, it also provides some reason for capacitists to prefer the knowledge option. In this way, it also contributes to attaining the perhaps central aim of the remainder of this paper, which is to mount a case that capacitists will do well to go for the knowledge option.

To see why Schellenberg’s analysis remains unsatisfactory, let’s return to the Gettier case in which you look at a lump of gold with a coating of fake gold that is indiscriminable from real gold by perception alone. What I’d like to ask is exactly what the capacitist analysis of perceptual knowledge predicts here. To be more precise, I want to consider the analysis independently of the decision as to whether the epistemic good in terms of which capacities are explained is knowledge or true belief. Instead, I want to consider it on its own terms, i.e. along the lines proposed by Schellenberg, according to which what matters is singling out particulars in the environment. Here it is once more:

Subject *S* has perceptual knowledge that *p* if and only if *p* is true, *S* employed a capacity to single out what she purports to single out, and *S*’s mental state has the content it has in virtue of *S* having successfully employed her capacity to single out what she purports to single out. (Schellenberg 2018, p. 206)

In the Gettier case at hand, did you employ a capacity to single out what you purport to single out? Clearly yes. You purport to single out gold and employed a capacity to recognise gold. And does your belief that the lump is gold have the content it has in virtue of you having successfully employed your capacity to single out what you purport to single out? The answer to this question turns on what it takes to successfully employ your capacity to single out what you purport to single out. Now, you purport to single out gold. What’s more, you successfully do single out gold. After all, the lump you are looking at is a lump of gold. So, you did successfully employ your capacity to single out what you purported to single out. The last question then is whether your belief has the content it has, i.e. that the lump is gold, in virtue of you having successfully employed your capacity to single out what you purported to single out, i.e. gold. Again, the answer here is clearly yes. But in that case, the capacitist analysis predicts that your belief that the lump is gold qualifies as knowledge.^6^

We are now in a position to see the third critical result of this paper: Schellenberg’s analysis of perceptual knowledge runs into the Gettier problem even if it is considered on its own terms, i.e. independently of the question of whether epistemically good capacities are to be analysed in terms of knowledge or true belief. Of course, this does not show that it is impossible to provide a capacitist analysis of perceptual knowledge. However, it is a major setback for capacitists who want to hold on to the idea of giving a reductive analysis of knowledge. After all, they will now have to go back to the drawing board and develop a new analysis of perceptual knowledge. By the same token, it provides at least some reason for capacitists to favour the knowledge option for the key choice. After all, the central drawback of this option is that it the capacitist analysis of perceptual knowledge will have to go. Once it is clear that there is independent reason to think that the analysis doesn’t work, it is much less clear just how much of a drawback this really is.

## How to make the key choice

In this section, I want to sketch one promising way forward for capacitists that draws on some of my own recent work (Kelp, 2021a, b). To begin with, let’s return to the key choice between knowledge and true belief as the epistemic good in terms of which good epistemic capacities are explained. How to make this choice? In what follows, I will develop one promising answer to this question.

Recall that Schellenberg distinguishes among four approaches to epistemological theorising: capacitism, knowledge first epistemology, evidential internalism, and reliabilism. I have recently developed an alternative with starts epistemological theorising with inquiry or the activity of finding things out.^7^ Inquiry is an activity with constitutive aims and norms. For present purposes I will focus on inquiry into specific whether questions (henceforth just ‘inquiry’), for instance, the question whether there is milk in the fridge or whether the Prime Minister will resign before the end of the month. The constitutive aim of inquiry can be characterised in a lightweight manner in terms of settling these questions. For instance, inquiry into whether there is milk in the fridge aims at settling the question as to whether there is milk in the fridge (Kelp, 2021a, b).

Now, one key theoretical idea of this view is that activities with constitutive aims and norms constitute normative domains and that the constitutive aims are valuable for their own sake, relative to these domains. For instance, chess is one example here. The constitutive aim of chess is to checkmate one’s opponent. Accordingly, checkmating is the for-its-own-sake good in the normative domain constituted by chess. Likewise, since inquiry is an activity with constitutive aims and norms, it constitutes a normative domain. And since settling questions is the constitutive aim of inquiry, it is the for-its-own-sake good in the normative domain constituted by inquiry (Kelp, 2021a, b).

A second key theoretical idea is that the epistemic domain just is the normative domain constituted by the inquiry. On this view, then the constitutive aim of inquiry is the for-its-own sake good in the epistemic domain. In this way, question settling is the for-its-own-sake epistemic good (Kelp, 2021a, b).

Now, I pointed out that the constitutive aim of inquiry can be characterised in a lightweight manner in terms of question settling. At the same time, there is a lively debate on the aim of inquiry in which a variety of substantive claims about the aim of inquiry are defended. Unsurprisingly, the most popular candidates here are knowledge (e.g. Kelp 2021a, b, 2014, Millar, 2011, Williamson, 2000) and true belief (e.g. Kvanvig, 2003, Lynch, 2004).

With these points in play, here is why the inquiry-based approach to epistemological theorising holds out hope for capacitists to arrive at a motivated way of making the key choice. The approach makes room for an argument-based debate over what the relevant for-its-own-sake epistemic good is. After all, on this view, the answer will turn on whether knowledge or true belief is the aim of inquiry. If true belief is the aim of inquiry, then true belief is the for-its-own-sake epistemic good. In that case, we have reason to think that epistemically good capacities are capacities to produce true beliefs. And while the prospects of an account of justification in terms of knowledge may look dim at this stage, the possibility of a reductive analysis of knowledge remains very much on the table (even if it can’t be quite as Schellenberg envisages, as per Sect. 5). In sum, capacitists will have reason to go for the true belief option when facing the key choice. In contrast, if knowledge is the aim of inquiry, then knowledge is the for-its-own-sake epistemic good and epistemically good capacities are capacities to produce knowledge. If is option is the right one, then we must abandon the hope of a reductive analysis of knowledge in terms of epistemically good capacities. At the same time, there is every reason to think that we will be able to give an account of justification in terms of knowledge. Capacitists will have reason to go for the knowledge option instead.

What comes to light is that, on the inquiry-centric approach, the key choice will come down to the question as to whether the aim of inquiry is true belief or knowledge. Crucially, this is a question we can (and indeed do) have an argument-based debate about. Note also that we do not have to rely on prior traditional or a knowledge first commitments, even when it comes to questions about epistemic normativity. On the contrary, we can approach the key choice with an open mind and let the chips fall as they may. This is the paper’s second constructive point.

## In favour of the knowledge option

In this last section, I will provide some reason for thinking that capacitists will do well to go with the knowledge option on the key choice. In a nutshell, this is because there is reason to think that knowledge is the aim of inquiry. While I have belaboured this point in considerable detail elsewhere (e.g. Kelp, 2021a, b, 2014) will here rest content with sketching one central positive argument for this claim.

The key idea is that agents in Gettier cases have justified true beliefs, but they do not succeed in their inquiries. In other words, agents in Gettier cases don’t attain the aim of inquiry. Here is one way to make this point. First, when we engage in activities with aims, we will be released from any commitment to attaining the aim we may have undertaken if we do indeed attain said aim. For instance, suppose you have undertaken some commitments to running a certain marathon. Crucially, when you have run that marathon, you are released from any commitment to run it you may have had. Now, since attaining the aim of an activity with an aim releases us from any commitment to attaining this aim, claims about the aim of inquiry will make predictions about when we are released from our commitments to attaining the aim of inquiry. In particular, the true belief account will predict that we are thus released when we have a true belief. In contrast, the knowledge account will predict that we will be released when we have knowledge.

Now, there is reason to think that prediction of the true belief account is mistaken, as cases like the following serve to show.

You are a geologist. I have hired you for two weeks to find out whether (D =) a certain mine that I am considering buying still has diamonds in it. Since I need to be in a remote location with no means of communication for the next two weeks, we agree to meet at the mine two weeks from now. You send your team of workers to the mine. After a day’s work, they bring you a sample of a deposit of stones they found. You run all available tests on a sample of the stones, all of which suggest that the stones are indeed diamonds. Based on this evidence, you come to believe that D. Since there is still a considerable amount of time before our meeting, you and your team pack your bags and get on the next flight home to spend time with your families. Meanwhile, I returned unexpectedly early from my trip to the breaking news that the seller of the mine had placed a deposit of fake stones in the mine that are so cleverly crafted as to be indistinguishable from real ones by currently available tests. What’s more, I also learn that the fake stones were placed exactly where you found the deposit and that you have since left to see your family. You are currently back home with your family and entirely unaware of the news. The final twist in the story is that, unbeknownst to everyone, there actually is a deposit of real diamonds in a hidden corner of the mine. (Kelp 2021a, p. 14)

In this case, you have a justified true belief that D, which falls short knowledge. The true belief account predicts that you are released from your commitment to inquiry into whether D. Crucially, this is the wrong result. In particular, you are not released from your contractual commitment to inquiring into whether D. If I find out about what’s going on, I may insist that you go back to work and fulfil your contract. Note that I do not need to negotiate a new contract with you. Rather, the old one is still binding. If so, you were not released from your contractual commitment to inquire into D. The true belief account makes the wrong prediction. In contrast, the knowledge account steers clear of this problem. After all, the case is a Gettier case, i.e. a case in which your belief falls short of knowledge. The knowledge account doesn’t make the wrong prediction. In this way, there is evidence that knowledge account is preferable to the true belief account (Kelp, 2021a).

To repeat, since on the inquiry-centric approach to epistemological theorising, the constitutive aim of inquiry is the for-its-own-sake epistemic good, the argument that knowledge rather true belief is the aim of inquiry converts into an argument that knowledge rather than true belief is the for-its-own-sake epistemic good. As a result, there is now independent reason for capacitists to go with the knowledge option. In this way, capacitists not only have a motivated way of making the key choice, but they also have reason to go for one option in particular rather than the other.

Before closing, I’d like to note that there is reason to think that Schellenberg’s own resources serve to bolster this point. To see this, suppose that Schellenberg is right in that the function of perceptual capacities is to single out particulars in the environment. If so, what it takes for perceptual capacities to be successful involves the singling out of a particular in the environment. What’s more, on the inquiry-centric view, when you form a perceptual belief that there is a dog ahead, say, via the employment of a perceptual capacity to recognise dogs, you conduct an inquiry into the question of whether there is a dog ahead.^8^ But it is hard to see how this inquiry could be successful unless the employment of the perceptual capacity to recognise dogs via which it is conducted is successful. After all, the inquiry is successful only if your perceptual belief that there is dog ahead qualifies as knowledge. At the same time, it is hard to see how you could acquire perceptual knowledge that there is a dog ahead unless you successfully singled out a dog, i.e. unless your employment of the perceptual capacity to recognise dogs was successful also. But, of course, you can arrive at a true perceptual belief that there is a dog before you via the employment of a perceptual capacity to recognise dogs without singling out a dog in the environment. After all, recall the case in which you are looking at a robot dog that looks just like a dog. In this case, what you are singling out isn’t a dog and, indeed, you have failed to single out a dog in the environment. By the same token, the employment of your perceptual capacity to recognise dogs has not been successful here. At the same time, your belief may very well be true. It may be that there is a dog ahead, perhaps hidden from view behind the robot.

In this way, capacitism provides the theoretical resources for a further argument that knowledge rather than true belief is the aim of inquiry. To be more precise, it is the capacitist account of perceptual success that does the heavy lifting here. On this view, perceptual success requires singling out a particular of the kind that the capacity is a capacity to single out. Crucially, Gettier cases serve to show that one can acquire a true perceptual belief via employing a perceptual capacity without having singled out a particular of the kind that the capacity is a capacity to single out. True belief is too weak a condition for perceptual success. And since inquiry via the employment of perceptual capacities will be successful only if the capacities are successfully employed, i.e. only if perceptual success is attained, we get the result that true belief is too weak a condition for success in inquiry. At the same time, it is easy enough to see that the knowledge view does not encounter the same problem. After all, Gettier cases are cases in which knowledge is absent. And that’s why Schellenberg’s view serves to bolster the claim that knowledge rather than true belief is the aim of inquiry. By the same token, it provides capacitists with further reason to go with the knowledge option. In this way, we have two further constructive points.

## Conclusion

This paper has taken a close look at capacitism, according to which epistemological theorising starts with capacities, which are explanatorily basic, and which aims to analyse other key epistemic phenomena in terms of capacities.

I have argued that capacitism faces a problem. Capacities can be epistemically good or bad and to get epistemological theorising off the ground we need to be able to tell the good ones from the bad ones. I suggested that the way to do this is in terms of epistemic goods, with the two candidates here being knowledge and true belief. I have also argued that the key choice isn’t entirely straightforward for capacitists. If they go with knowledge, the envisaged reductive analysis of knowledge is in jeopardy. If they go with true belief, the direction of explanation between knowledge and justification doesn’t work as envisaged and the Gettier problem starts looming.

I then provided reason to think that Schellenberg’s reductive analysis runs into the Gettier problem even if it is considered on its own terms. In this way, I provided not only an interesting critical result for capacitism but also at least some initial reason for capacitists to favour the knowledge option.

My central positive suggestion for capacitism here was to opt for an inquiry-centric approach to epistemology, which allows for an argument-based way of making the key choice. I also provided further reason for going with the knowledge option, including one reason provided by capacitism’s own theoretical resources. Of course, between the two options at issue in the key choice, the knowledge option is the one that will align capacitism more closely with knowledge first epistemology. In this way, the argument for the knowledge option converts into an argument for capacitists to align themselves more closely with knowledge first epistemology.

## Footnotes

1 Note that perceptual capacities may differ in this respect. After all, it is arguable that perceptual capacities are necessarily capacities to produce epistemic goods.

2 Couldn’t capacitists go for some other option? While this may, in principle, be possible, in practice, there aren’t any good alternatives. To see this, note first that true belief and knowledge are promising candidates. After all, functional entities such as capacities are good relative to some kind of good because they are good means to attaining this kind of good. But now note that the key debate over the central epistemic good is between traditionalists and knowledge firsters. Traditionalists hold that true belief is the central epistemic good. Justification is unpacked instrumentally as a good means to true belief, and knowledge is then analysed in terms of justified true belief and some anti-Gettier condition. To hold that true belief is the epistemic good in terms of which epistemically good capacities are analysed is just adopt a Schellenbergian version of traditionalist epistemology. On the other hand, knowledge firsters hold that knowledge is the central epistemic good rather than true belief. Knowledge firsters think that knowledge cannot be analysed (at least not in terms of justified belief) and want to analyse justified belief in terms of knowledge. To hold that knowledge is the epistemic good in terms of which epistemically good capacities are analysed is just to adopt a Schellenbergian version of knowledge first epistemology. One thing that both options have going for themselves is that they make epistemological and value theoretic sense. What about other options? Some have held that justified belief is the central epistemic good (Davidson, 2005; Feldman, 2002). This option doesn’t seem available for Schellenberg since she wants to analyse justified belief in terms of capacities. On the view we are considering, justified belief would be the epistemic good in terms of which capacities are analysed, which would preclude an analysis of justified belief in terms of capacities. What other options might there be? Certainty and understanding seem too demanding to be plausible candidates. We wouldn’t want to say that epistemically good capacities are capacities that have the function of producing certainty or understanding and that capacities that don’t fall short of being epistemically good. This becomes particularly clear once we look beyond the epistemology of perception. When it comes to capacities involved in testimonial and memorial knowledge, the prospect of being committed to the claim that these capacities fall short when they don’t produce certainty or understanding looks entirely unappetising. In this way, certainty and understanding would lead to an unduly restrictive view of the function of good epistemic capacities. Beyond this, I cannot think of any further worthwhile candidate for the central epistemic good in terms of which epistemically good capacities are to be analysed. Of course, I might just be missing something here. But, if Schellenberg does indeed have something else in mind, at this stage, it seems fair to leave it to her to tell us what exactly it is and to work out the corresponding alternative view.

3 Recall that I pointed out at the beginning of Sect. 2 that Schellenberg takes her view to be an alternative to knowledge first epistemology, internalist evidentialism, and reliabilism. I take this to be excellent evidence that she takes capacitism to be a general epistemological view. After all, it is hard to see how her view could be an alternative these views unless it was a general epistemological view. By way of further evidence, note that, in the same context, she gives a characterisation of the view that is not restricted to the epistemology of perception: “By contrast, capacity views treat capacities as explanatorily basic and analyze evidence, justification, and knowledge as a product of the capacities employed. So, on the first cluster of views, conscious mental states are explanatorily basic, on the second cluster knowledge, on the third reliability, and on the fourth capacities” (Schellenberg, 2018, pp. 187–188). Thanks to an anonymous reviewer for pressing me on this issue.

4 One might also wonder whether Schellenberg couldn’t retract capacitism as a general epistemological view and just hold on to capacitism for the epistemology of perception. Unfortunately, this route also does not look promising. To see this, recall that Schellenberg claims that perceptual knowledge admits of analysis in terms of capacities, and let’s ask whether other kinds of knowledge also admit of analysis. If the answer is no, the view is clearly theoretically highly unsatisfactory. After all, why should it be that we can analyse perceptual knowledge but not, say, inferential or testimonial knowledge? What’s more, the fact that an analysis of a specific kind of knowledge doesn’t generalise to other kinds of knowledge is widely regarded as a problem for the analysis in question, as the case of the causal theory of knowledge and a priori knowledge clearly indicates. Finally, the view will be at a disadvantage vis-à-vis rival epistemological approaches that do not face this generalisation problem. Since in what follows I develop one such view (for capacitists), the prospects of making a negative answer work are increasingly dim. On the other hand, if the answer is yes, there is still trouble. One reason for this is that it would still be unsatisfying to say that perceptual knowledge and other kinds of knowledge admit of analysis in terms of perceptual etc. capacities, but there simply isn’t a general analysis of knowledge in terms of capacities. Another reason is that it is just implausible that there are analyses at the level of specificity that Schellenberg offers for perception for all other kinds of knowledge. The clearest example here is testimonial knowledge. While our perceptual capacities may well have a very specific function such as the function to single out and discriminate particulars in the environment, the same does not appear to be true of testimonial capacities. In fact, there is little hope of identifying a function of our testimonial capacities that is more specific than the acquisition of true belief/knowledge from the say-so of others.

5 Couldn’t Schellenberg avoid this problem by holding that perceptual capacities have the function to single out only low-level properties? No. As a first observation, note that Schellenberg wants to remain neutral on this issue. But, of course, this response rules out neutrality. Second, and more importantly, it is easy enough to construct a version of the case that involves only low-level properties. Consider: Due to unusual lightning conditions, a red object appears green to normal human perceivers. At the same time, unbeknownst to you someone fitted you with contact lenses that invert your colour spectrum such that things that appear green to normal human perceivers appear red to you, and you form a belief that the object before you is red (alternatively: unbeknownst to you, you are looking through a window that inverts your colour spectrum; alternatively: unbeknownst to you, someone has, perhaps temporarily, messed with your colour vision such that everything that appears either red or green to normal human perceivers will appear red to you).

6 According to Schellenberg, perceptual capacities are capacities to discriminate and single out individuals. At the same time, discrimination makes no appearance in her definition of perceptual knowledge. Couldn’t the looming Gettier problem be avoided once discrimination is properly factored into Schellenberg’s definition? After all, it is tempting to describe the problem with your belief in terms of discrimination. You are unable to discriminate a case in which the lump is gold from a case in which the lump is fake gold. That’s why you lack knowledge. Even if this route may be viable in principle, there is reason to think that it isn’t an option for Schellenberg. The reason for this is that she is clear that by discrimination she means ‘material discrimination’ which is a relation that occurs between agents and two distinct actual mind-independent particulars (Schellenberg, 2018, p. 28). Since the case in which the lump is fake gold is non-actual, the fact that you do not discriminate between the actual case and this case is of no consequence for whether you successfully materially discriminate the lump of gold. What’s more, it is not an accident that Schellenberg goes for material discrimination rather than its counterfactual cousin. The reason for this is that she wants to use perceptual capacities to understand not only issues in epistemology but also in the philosophy of mind. In particular, she wants to use perceptual capacities to understand perception. Crucially, Schellenberg is clear that “there is no reason to think that modal appreciation [of the kind is required by counterfactual discrimination] is constitutive of perception” (Schellenberg, 2018, p. 38). This is why, ultimately, this move will be unappealing for her.

7 In what follows, I will state the view in terms of inquiry. Note that for this view to be plausible, we’ll need a weak account of inquiry according to which cases of automatic belief formation count as inquiry. Those who find this implausible can always substitute ‘activity of finding things out’ for inquiry.

8 Recall that I am working with an inclusive account of inquiry. For those who want to resist it, note that I am in any case engaging in the activity of finding out whether there is a dog in the field before me.
